# DRAGen – A deep learning supported RVE generator framework for complex microstructure models

**DOI:** 10.1016/j.heliyon.2023.e19003

**Published:** 2023-08-09

**Authors:** Manuel Henrich, Niklas Fehlemann, Felix Bexter, Maximilian Neite, Linghao Kong, Fuhui Shen, Markus Könemann, Michael Dölz, Sebastian Münstermann

**Affiliations:** Integrity of Materials and Structures, RWTH Aachen University, Intzestraße 1, Aachen, 52064, Germany

**Keywords:** Representative Volume Elements, Crystal plasticity, Microstructure modeling, Deep learning, Microstructure design

## Abstract

In this study an improved version of the Discrete RVE Automation and Generation Framework, also called DRAGen, is presented. The Framework incorporates a generator for Representative Volume Elements (RVEs). Several complex microstructure features, extracted from real microstructures, have been added to the generator, to enable it to generate RVEs with realistic microstructures. DRAGen is now capable of reading trained neural networks as well as .csv-files as input data for the microstructure generation. Furthermore, features such as pores and inclusions, martensite bands, hierarchical substructures, and crystallographic textures can be reconstructed in the RVEs. Besides the features, the functionality for different solvers was introduced. Therefore, the code was extended by modules for the generation of Finite Element (FE) and spectral solver input files. DRAGen now has the ability to create models for three powerful multiphysics frameworks used in the community: DAMASK, Abaqus and MOOSE. The evaluation of the features, as well as the simulations performed on sample models, show that the new version of DRAGen is a very powerful tool with flexible applicability for scientists in the ICME community. Also, due to the modular architecture of the project, the code can easily be expanded with features of interest. Therefore, it delivers a variety of functions and possible outputs, which offers researchers a broad spectrum of microstructures that can be used in microstructure studies or microstructure design developments.

## Highlights

• Introduction of refactored RVE generator.
• Presentation of optically accurate modeling of complex microstructure features.
• Quantification of complex microstructure features and validation of models.
• Reconstruction of pores, inclusions, martensitic bands, pronounced textures and hierarchical substructures.

## Graphical abstract

**Figure:**
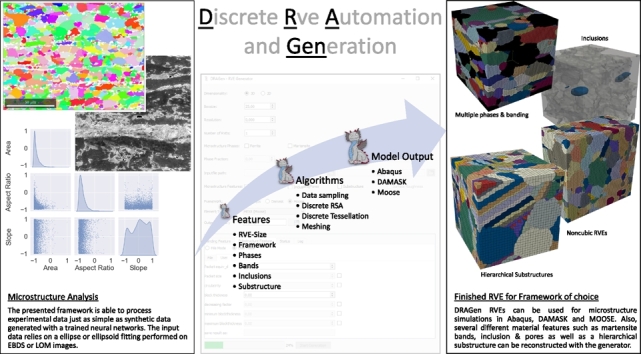

## 1 Introduction

The global climate crisis confronts many industrial sections with major challenges. To meet the 1.5 ^∘^C goal which was set in the Paris treaty, companies have to decrease their CO_2_ emissions significantly which is the most challenging task to current and future generations if the currently high living standards are kept at the same level [1], [2], [3]. According to Allwood and Cullen [4] in the material sector three main strategies exist to reduce CO_2_ emissions:

• Reducing the material usage through efficient use of materials in components.
• Improving the efficiency of material usage by reduction of the off cuts.
• Increasing the lifetime of components combined with a proper recycling strategy.

According to the thyssenkrupp Steel Europe AG [5] the company alone causes 2.5% of Germany's CO_2_ emissions. This shows the huge potential of reducing emissions by using less material. Thus, the first strategy is highly interesting for materials engineers and scientists in the field of steels and other metallic materials. The strategy depends on further developments of computational methods describing the material behavior in a realistic manner. Once these methods can be used to predict the integrity and performance of materials reliably it is possible to select the perfect material for a specific application from a pool of already existing materials and even reduce the amount of material in the component. One possible approach to the accurate description of metallic materials like steels is the geometric modeling of the microstructure and the performance of simulations with these models [4].

In modern steels, depending on the manufacturing process multiple phases appear in the final microstructure. Each phase contributes its own characteristics to the microstructure. This effect is currently used to tune mechanical properties of the material [6]. A combination of multiple phases like ferrite and martensite leads to high strength steels with good formability, however these microstructures show increasingly complex grain shapes and morphologies or banded martensite structures. Many steels in the family of Advanced High Strength Steels (AHSS) contain several coexisting phases with these features as it is the case in dual phase steels (DP steels) or complex phase steels (CP steels) [7], [8], [9].

Other steel grades are purely martensitic or bainitic and contain complicated hierarchical substructures. The hierarchy of these substructures is a result of the transformation from austenite to martensite or bainite. Therefore, in the final microstructure Prior Austenite Grains (PAGs) can be detected. According to Morito et al. [10], Kitahara et al. [11] and Bhadeshia [12] these PAGs contain so called packets which again contain blocks. The reconstruction of these substructures can be performed using the algorithm from Niessen et al. [13] made available in the Matlab Toolbox MTEX (Version 5.8.0). Any of the mentioned complex microstructure features can lead to damage mechanisms that cannot be predicted on a macroscopic scale. Some of these effects are e.g. martensite fracture, microstructural notch effects or strain localization and pore incubation due to high strength gradients in multiphase materials [14]. An experimental parameterization of microstructure's to an extent which is necessary to investigate the influence on the mechanical properties of modern steels is impractical since the microstructure parameters influence each other as well as the mechanical behavior. Meaning parameter A e.g. the aspect ratio may change as well while experimentally varying the microstructures parameter B e.g. the grain size. Therefore, the more parameters identified in the microstructure, the more complex it becomes to vary only one parameter at a time [15].

A solution to this problem can be found in computational methods. To resolve the influence of each parameter on the macroscopic behavior numerical microstructure models also called Representative Volume Elements (RVEs) have been found to be a powerful tool [16]. The use of those models allows to vary each parameter at a time while not touching the other parameters which again allows a differentiated evaluation of each parameter. Such kind of study was performed by Gillner and Münstermann [17] and Gillner et al. [18]. A common method is to combine the geometric material models with a constitutive material model such as the crystal plasticity model. Several different approaches for calculating the single crystal behavior have been developed in the past decades. A substantial summary covering these models was published by Roters [19]. These material models describe the single crystal behavior with phenomenological equations that were derived and motivated by the mechanical behavior of crystals or by physically motivated equations. The model is then applied to each grain representing volume in the RVE. The combination of the geometric and the material model allows a stress analysis on microstructural scales. The accuracy of said stress analysis is dependent on both parts of the model. The description of the material behavior needs to be realistic and well calibrated but also the geometric description of the polycrystal must be reconstructed with great care. In many studies the focus lies on the first of these two parts and a great effort has already been made to describe the single crystal behavior as well as possible [20], [21], [22], [23].

Yet, most studies only consider the material modeling as the important part. Considering complex microstructures as they are given in most modern and high strength steels the geometric features should be considered as evenly important particularly when the scattering of damage initiation or damage mechanisms in general is to be studied. Thus, investigations on the material behavior of a polycrystal with a geometrical over simplified model cannot lead to accurate results, nor can they describe the effect of microstructural features that were eradicated by the simplification of the model. So, to achieve the overall goal of accurately predicting the materials behavior it is of high importance that these microstructure models represent reality as close as possible while still containing a certain degree of statistical scatter. [15], [24]

Motivated by this, a previous study by Henrich et al. [25] presented a basic structure for microstructure modeling with complex features. Additionally, Pütz et al. [26] published an algorithm that uses Artificial Neural Networks (ANNs) in order to reproduce the characteristics of any microstructural feature of interest. A further development of this method was published by Fehlemann et al. [27]. In addition to the representation of different features the microstructure models must be created in a format which researchers can easily use for simulation frameworks such as Abaqus, DAMASK or MOOSE. Each of these frameworks has its own characteristics with great advantages but also some disadvantages.

DAMASK e.g. is mainly built for a spectral solver and it is rather difficult to run macroscopic simulations with non periodic models. For periodic microstructure models however, this is a great framework since the spectral method is relatively fast offering the possibility to create some statistics over multiple simulations which would be much more time consuming in other frameworks [22]. Abaqus in comparison comes with a whole toolbox for technical drawings and it is possible to build complex geometries within the software and apply many different material types to the model. It is also possible to implement user defined materials by using so called user subroutines. However, there are some limitations regarding the physical coupling of models. There is only one nonlocal field available within the standard subroutines which is occupied by the temperature. In case the user wants to calculate other effects in the material depending on nonlocal effects such as a damage field or hydrogen diffusion, Abaqus does not offer a out of the box solution. Another issue about Abaqus is that it is not available without any financial charge. Also RVE simulations with Finite Element (FE) solvers are slower than those performed with a spectral solver [28]. MOOSE on the other hand just like DAMASK is an open source framework, using however an FE solver. MOOSE allows implementing and combining different physical and phenomenological models relatively easy and also offers a large variety of pre-build models for e.g. tensor mechanics or phase field theory. While this framework offers the most flexibility in terms of material modeling it also embodies the most time consuming option in terms of CPU time. Thus, it must be said that all three option have very good arguments on their side which makes them very attractive for material scientists.

To get the best possible investigations out of all three frameworks our team built a new version of the DRAGen RVE generator introduced by Henrich et al. [25] with all the above mentioned features and functionalites, introduced in this study. These developments contain all the important microstructure features mentioned above, as well as an easy to use output for the mentioned frameworks. All supported features and functionalities of DRAGen v.1.0 are listed below:

• Representation of multiple phases,
• Representation of non-metallic inclusions and pores,
• Representation of martensitic bands,
• Hierarchical substructure generation,
• Generation of non-cubic RVE volumes,
• Texture reconstruction,
• Transformation of the discrete grid into a FE Mesh with smooth grain boundaries,
• Graphical User Interface,
• Possible outputs files are DAMASK Spectral input files, Abaqus input files and MOOSE input files.

## 2 State of the art

This section provides a short overview on the two very prominent RVE generators Dream.3D and Neper as well as a short review of the work of Pütz et al. [26] to give a better understanding of the data types DRAGen v.1.0 is now able to process. Also, the algorithms used in the main body of DRAGen v.0.1 and introduced in Henrich et al. [25] are briefly discussed.

### 2.1 Dream.3D and Neper

The two most common publicly available RVE generators in the poly crystal community are Dream.3D and Neper. Both generators are quite powerful and have a large community of users. Dream.3D was initially developed for the reconstruction of 3D Electron Back Scatter Diffraction (EBSD) data from serial sectioning images [29]. Within the software it is possible to define so called pipelines which are a combination of imaging and data processing filters. These pipelines are used for the analysis of experimental data or the generation of synthetic data with respect to phase ratio, grain shape and texture [29]. Even though Dream.3D is designed to be extended by new features constantly, it was found to be rather difficult to implement banded structures of a secondary phase.

Neper on the other hand is based on a Voronoi tessellator offering the possibility for multiscale tessellation which can be used for materials or phases with hierarchical substructures such as martensite or bainite. The Voronoi tessellation leads to grains strongly reminiscent of spheres. However, grains in real microstructures often have ellipsoidal shaped or even concave shaped grains and cannot be represented by spherical objects. Also, for the multiscale tessellation only two levels are available, meaning it is not possible for an RVE generated with Neper to represent children and grandchildren of a grain object. To put it simple, Neper can only reconstruct PAGs and packets or packets and blocks but not all three at the same time. If microstructural effects are supposed to be studied in a realistic manner, yet this is inevitable. [11], [30], [31]

Both, Dream.3D and Neper have several options for the output format which allows the user to generate RVEs for different applications such as Abaqus or DAMASK. When it comes to MOOSE however none of them actively supports the ExodusII data format which is the chosen standard in the MOOSE framework. Yet, MOOSE is able to read the vtk data format which is an option in both generators. Yet, the user does experience some drawbacks with the vtk format. MOOSE relies on node and element sets for the assignment of boundary and loading conditions. When done correctly these node and element sets can be stored in the ExodusII mesh file. However, this option is not available for the vtk format. So, if users have to use vtk meshes in MOOSE the node and element sets also have to be defined manually. [32]

### 2.2 Generation of statistically representative input parameters

In order to extract the microstructure information usually metallographic images e.g. Light Optical Microscopy (LOM) images or images from EBSD measurements are used. The advantage of EBSD-images is clearly the information about the orientation of each grain. Besides that, the same method can be applied on LOM and on EBSD images: An ellipse fit for each grain provides the necessary information for the RVE generation. These ellipse fits can be performed using several freely available software packages such as MTEX [33], the image software Fiji [34] or similar software packages like scikit-image available for python [35].

However, there are two main issues in generating realistic microstructure models on a statistical basis. First, the measured data usually comes in only two dimensions since they are extracted from 2D-images from an EBSD measurement or an LOM image. Second, microstructures contain several characteristics that all depend on each other and these interdependencies between e.g. the grain size and the grain shape or the grain size and the grain orientation cannot be considered as isolated distributions. If done so, the final data set will contain combinations of grain characteristics that never appear in the underlying experimental data. [26]

The only way to truly solve the first issue is to collect 3D-data via serial sectioning methods or projection based imaging with a rotating sample [6]. Since these methods come with huge experimental efforts the third dimension for all the following data sets is estimated from 2D-images in different cross sections. However, all presented approaches are capable of conducting 3D-data sets meaning all developed concepts are designed to eventually work on real 3D-data sets. [26]

**Figure 1:**
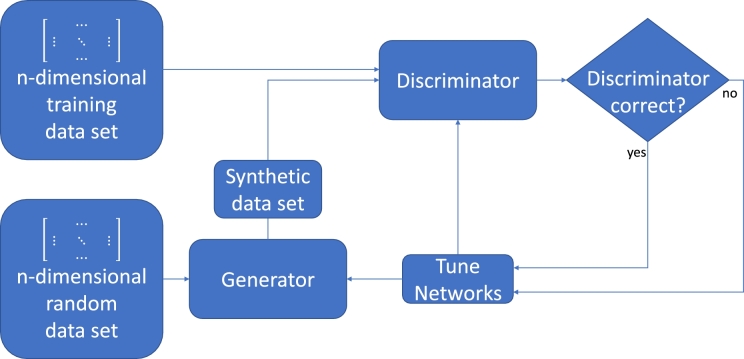
Schematic illustration of the WGAN architecture according to [36].

The second issue was tackled in a preceding publication from our group [26]. To determine the correct multidimensional distributions and generate synthetic data from these Pütz et al. used a Wasserstein Generative Adversarial Network (WGAN). This deep learning network architecture uses feed forward networks competing against each other, a so called generator network and a discriminator network. The idea is that the generator network takes a random data set, manipulates it according to the weights in its neurons and passes it on to the discriminator which at the same time receives training data from the data set that is supposed to be reconstructed. To put it simple, the discriminator must now decide if the presented data set is a real or a synthetic one. Both discriminator and generator get a feedback of the result and for both networks the weights of the neurons are optimized for the next epoch. This way the generator tries to trick the discriminator into declaring a synthetic set as the real data set. This procedure is repeated until the generator reaches a sufficient accuracy. Once this is the case the generator network is stored in its current stage as well as the training data. Both objects are wrapped in a so called .pkl-file which is a serialization standard within python. This file can later on be unwrapped to use the generator to generate synthetic data. Since the WGAN only works with values between −1 and 1 the training data is needed to denormalize the synthetic data, which is why it was wrapped together with the generator into the .pkl-file [26]. Fig. 1 shows a schematic illustration of the WGAN architecture.

**Figure 2:**
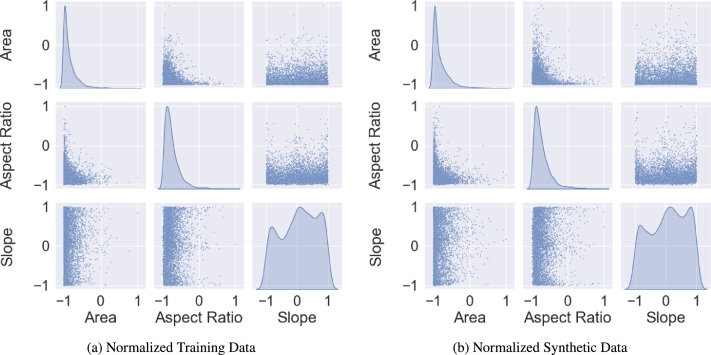
Comparison of the normalized data set used as training data (a) and the normalized synthetic data (b) generated by the WGAN algorithm.

Regarding the microstructures, each dimension in the data set represents one parameter in the microstructure to be reconstructed e.g. the grain size or aspect ratio of the grains. Any parameter that can be measured and quantified with a number is a possible candidate for reconstruction in a synthetic data set. Fig. 2 exemplarily shows a pair plot of several microstructure parameters that were used for a training of the generator. The pairplot in Fig. 2a shows the training data while Fig. 2b contains the pairplot with the resulting synthetic output of said generator. For both data sets the normalized data is depicted to keep the axis labeling plain and simple. [26]

In DRAGen the WAGAN is responsible only for the generation of input data and has no further functionality in the generation process of the RVE. The great advantage of the WGAN approach is however, that after the generator was trained an unlimited amount of input data can be generated by the WGAN. Meaning, each generation from the network replaces a complete EBSD measurement of the material. This becomes particularly important when several RVEs have to be simulated for the same material, as it is the case for studies like those performed by Gillner et al. [37]. If instead of the .pkl-file a fixed experimental data set from a .csv-file is used for the generation of multiple RVEs, the same grain from that data set may be used multiple times. In general the .csv-file should contain a much larger number of grains than what actually fits in the chosen RVE volume. DRAGen randomly selects grains from this file until the total grain volume equals the chosen RVE volume. Since the selection follows a uniform distribution the material dependent distributions of the microstructure are not changed. It is however highly important that the data in .csv-file represents all distributions correctly. The .pkl-file from containing the WGAN generator on the other hand produces a new set of grains before the generation of each single RVE, which makes it very unlikely that the exact same input configuration for one grain will appear in multiple RVEs. Also, every new set of data follows the distributions the generator was trained on. Therefore, the statistics is always considered correctly.

Eventually the above mentioned .pkl-file can be used for further processing with the DRAGen RVE generator.

### 2.3 Structure of original DRAGen algorithm - DRAGen v.0.1

The plain DRAGen module introduced in [25] relies on two main algorithms, called modules, combined in a generation package.

• Discrete Random Sequential Addition (DRSA) algorithm
• Discrete tessellation algorithm

When using DRAGen v.0.1. users are required to generate their own set of input data. In this version DRAGen did not offer any generation algorithms for input data. In order to generate the microstructure models, the data of grains and features had to be stored in a .csv-file. It is assumed the data has the form as it is shown in Table 1. It is important to mention that the values of the ellipsoid raddi are linked to the cartesian coordinate system. Meaning, at an angle $\alpha =0^{\circ }$, *a* is the ellipsoid radius in *x*-direction, *b* in *y*-direction and *c* in *z*-direction and *α* describes the angle between the radius a and the *x*-axis. If a value for one of the variables is not given, DRAGen will assume the default value for it. So far only the basic grain structure is considered but no microstructure features. It is later shown, that the texture can also be applied within this data set. If there are multiple phases, a data file must be provided for each phase separately [25].

**Table 1:** Required parameters in the .csv-file for RVE generation.

	a	b	c	*α*	*φ*_1_	Φ	*φ*_2_
**Description**	ellipsoid radius	ellipsoid radius	ellipsoid radius	grain slope x-y-plane	euler angle	euler angle	euler angle
**Relevance**	mandatory	optional	optional	optional	optional	optional	optional
**Default**		a	a	0	random	random	random

The first module is a DRSA algorithm. A regular RSA algorithm can be used to randomly place hard spheres in a given volume and is originally an analytic method. Meaning the algorithm analytically avoids intersections of the spheres by calculating the exact distances of the sphere centers. To gain the best packing density the placing sequence should be sorted by their size, starting with the largest one [38]. DRAGen uses a slightly different but very powerful approach. Before placing any spheres the volume itself is discretized into a grid. When placing a sphere a grain ID is stored on each occupied grid point. Whenever the algorithm attempts to store a second grain ID on a grid point that means there is an intersection and placing at this location is forbidden. This approach has one major advantage: it does not depend on spheres. Any given shape can be used to be placed in the volume. Therefore, from now on ellipsoids will be considered. To ensure a good packing density the ellipsoids volume is reduced before the placing process and its original volume is stored in a list for later usage. While the original RSA algorithm cannot make sure ellipsoids are being packed as dense as possible and also do not intersect, the discrete version does. As mentioned before, for modern steels it is important to be able to also use ellipsoidal shapes [25].

Once all grains are placed in the volume, the next module takes over which gradually fills up the empty volume between the ellipsoids. In this algorithm the ellipsoid radii grow one voxel at a time. The order in which the ellipsoids grow is mixed after every step. Therefore, the growth of ellipsoids can be considered simultaneous. While all ellipsoids are growing, their size is being monitored. Once an ellipsoid reaches its original volume, stored in the list mentioned above, it is neglected for further growth. The simultaneous growth combined with the growth monitoring ensures that the grain size distribution from the input data is not changed significantly throughout the generation process of the RVE [25].

## 3 Further development of DRAGen

The final product of DRAGen v.0.1 is a grid object with grain IDs and phase IDs. This grid object can be further processed for calculations with the DAMASK spectral solver rather easily. For calculations with FE-Solvers no solutions were offered so far. Also the implementation of further microstructure features was rather suggested than solved. In the following sections, methods and functionalities of DRAGen v.1.0 are introduced. The prototype for a Graphical User Interface (GUI) is shown in Appendix A together with a brief description of its functionalities. This GUI is intended to make the use of DRAGen as simple as possible and to give scientists with little to no programming experience access to microstructure models with complex features. Table 2 shows a short overview of all currently available features incorporated in DRAGen and also in which version they have been introduced.

**Table 2:** DRAGen versions with its features.

feature	version 0.1	version 1.0
Code Architecture	Lists and Sets	Arrays
Finite Element Models	N.A.	Available
Grain size distribution	Available	Available
Grain shape distribution	N.A.	Available
Grain slope distribution	N.A.	Available
Number of different phases	2	4
Banded structures	Available	Heavily improved
Crystallographic texture	N.A.	Available
Hierarchical substructures	N.A.	Available

### 3.1 General changes in code architecture

The basic structure of DRAGen v.1.0 is build on the previous version. However for the modeling of further features such as banded microstructures and substructures some new modules had to be added. Also for building the microstructure models according to the desired solver framework new modules had to be written. At the time of publication the whole project is build in Python 3.7. Later versions are being constantly supported and the latest available tools for data processing and meshing techniques were used. Next to the development in the DRAGen code also the code for the WGAN was reviewed and updated. These changes have been published by Fehlemann et al. [27]. The changes in the WGAN code led to a more efficient reconstruction of RVE input parameters which makes it now possible to also consider the materials texture and also a variety of multiple materials within one network. The final version of the WGAN code was added to the DRAGen Code as a so called InputGenerator package.

**Figure 3:**
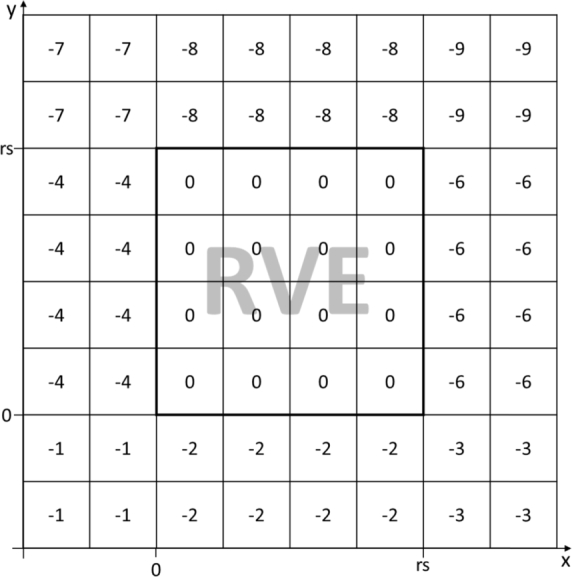
Boundary indices 2D.

**Figure 4:**
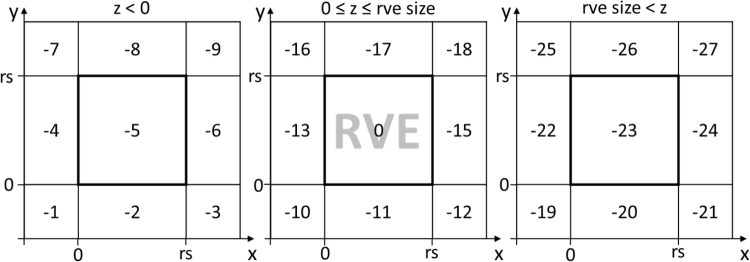
Boundary indices 3D.

The major update in the DRAGen code was switching from sets and lists to numpy arrays [39] and pandas data frames [40]. Both rely on very powerful c++ libraries and enable extremely efficient algorithms. Also the possibility for data management of the IDs and coordinates used in the DRAGen code is much more flexible using these data formats. Due to the changes of the data format a refactoring of most functions from version 0.1 was necessary. During the DRSA ellipsoids are being placed into an array as shown in Fig. 3. The cells containing zero values represent the RVE volume the outside areas are indexed with negative numbers starting at −1. When placing an ellipsoid, the algorithm basically overwrites the cell indices with a grain ID. If however, any one of the cell indices to be overwritten is a value greater than zero a collision is detected and a new location for the grain is chosen. If any one of the cell indices is less than zero it means the grain was partly placed outside the RVE volume and the periodicity monitoring function is triggered. This function moves the involved cells to the opposing side of the volume. To identify on which side the grain left the RVE volume the boarder indices are assigned a number according to its location. This indexing is shown in Fig. 3 for the 2D-case. All array cells in the location x<0 and y<0 are indexed with a negative 1 all cell arrays at 0<x<rve size and y<0 are indexed with a negative 2 and so on. For the 3D-case it works exactly the same yet a third dimension has to be considered which is why there are 26 areas outside the RVE volume. This scenario is shown in Fig. 4. Once any point of an ellipse or ellipsoid is placed outside of that volume, it can be checked which boundary index this cell of this point contains and with this information the numpy roll function can be used to roll this very point to the opposite side of the RVE volume in order to keep everything periodic. E.g. if an ellipse in 2D is placed on the right side of the RVE volume all points of the ellipse overlapping with the boundary cells are detected as ellipse cells with an index of −6. This means they will be rolled to the left by exactly the number of cells one RVE grid contains along the concerning direction. In the case of Fig. 3 this would mean by four cells, since the RVE in the figure is a 4×4 grid.

Once all grains were placed, the volume filling starts. This module has been updated to numpy arrays and pandas data frames as well and uses the same collision logic as the DRSA module. The periodicity during the tessellation is monitored with the same function as in the DRSA algorithm. Both, the update to pandas data frames and numpy arrays as well as the use of the new periodicity function leads to a much better efficiency and stability in the code.

### 3.2 Generation of finite element models

In order to use the DRAGen models in an FE framework such as Abaqus or MOOSE more modules for DRAGen were needed. In a first step the python package for vtk operations PyVista was used to transform the grid into an FE-Mesh with exactly one cubic element per grid point. Afterwards, an empty mesh which matches the grid exactly is available. However, so far the RVE mesh does not contain any grain information. Fortunately PyVista offers the option to map numpy arrays directly on the mesh cells. This option is used to map all the grain and phase IDs from inside the RVE volume numpy array onto the RVE mesh. The information about the grain and phase ID is stored in user defined variables after the mapping which are available on each element. With these operations a mesh object is generated which can be used for further processing [41]. Since all elements are cubes the grain boundaries are shaped like staircases. When stress distributions over the microstructures are considered these staircase shapes lead to unrealistic stress concentrations. Therefore, the boundaries have to be smoothed for a realistic stress analysis. For this purpose the laplacian smoothing also offered by the PyVista package was used. It can simply be called with the command *surface.smooth(n_iter)*. Where *surface* is the PyVista object containing the surface information of the grain and *n_iter* is an integer defining the number of iterations used for smoothing. In this study the default value was set to n_iter=250 iterations. Higher numbers were found to generate bad shaped meshes and lower values still contain many edges that tend to look unrealistic. In case the user needs a different element type such as C3D4 elements which are tetrahedral elements. This can be done right before the smoothing part. In this case, the TetGen package, which can also be called via PyVista, is used [42]. This package contains a tetrahedralization function which is able to tetrahedralize triangulated manifold surfaces. In order to use this package the surface object from PyVista is first triangulated and then tetrahedralized.

After the smoothing process the mesh object needs to be prepared for the framework of interest, either Abaqus or MOOSE. PyVista has access to the meshio library which is able to write Abaqus input files [43]. This option is used when Abaqus is requested. However the Abaqus input file from meshio only contains the node and element information. So, additionally the material sections have to be defined. This is done in an additional function within DRAGen. The DRAGen output for Abaqus is an .inp-file optimized for a UMAT-User-Subroutine implemented by Sharaf et al. [44]. It uses a usermaterial with two constants: the grain ID and the material type ID. These two numbers are used to read the grain orientation and the CP-Parameters from two separate files. If DRAGen users need a different definition for their usermaterial in abaqus this can easily be changed in the source code of DRAGen. While all the definitions for section assignments and the periodic boundary conditions can stay the same and do not have to be touched.

In MOOSE on the other hand the standard input file for meshes is the ExodusII format. ExodusII is a file format for FE meshes build on netCDF, developed by the Sandia National Laboratory [45]. Since the python API for Sandias ExodusII format is rather difficult to install and not straightforward and the meshio ExodusII output failed in generating a working mesh, an own mesher was written using the netCDF package available for python. This ensures that all requirements for the meshing process can easily be installed via the pip package installer in python and still provides the opportunity to generate RVEs in the ExodusII format. During the meshing process, all necessary node sets with the names “front”, “back”, “top”, “bottom”, “left” and “right” are automatically defined so they can be called in the MOOSE input file to define any desired boundary condition.

### 3.3 Reconstruction of microstructure features in DRAGen v.1.0

**Figure 5:**
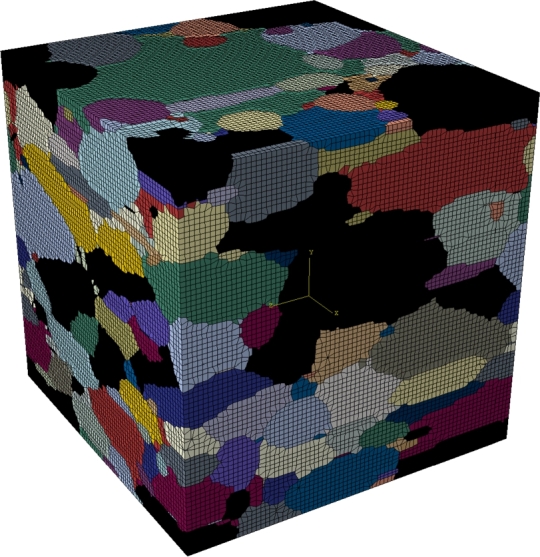
RVE of a Dual Phase steel. Martensite phase shown in black, colored grains represent ferrite with different crystallographic orientations.

In the following the most important features covered by the latest version of DRAGen are summarized. Including all features introduced in this study. At first the reconstruction of grain properties is discussed. The data for this discussion is derived from the RVE shown in Fig. 5. Afterwards, the features banding, substructure and texture will be explained in detail by presenting a example material and a descriptive modeling approach, since they represent the largest add-on to the code. Especially the features texture and hierarchical substructure are to be highlighted here, since the presented approach for these two features marks a novelty in the field of RVE generation.

Fig. 5 shows an RVE with a size of 50 μm^3^ and 77×77×77 elements. In total it contains 404 grains 301 of them represent ferrite and 103 represent the martensite phase.

In general, the whole study deals with pseudo 3D-data meaning the ellipses from 2D ellipse fits were extrapolated into ellipsoids by assuming the third half axis of each ellipsoid to be equal to the first one. This assumption was drawn for reasons of simplicity and due to the lack of actual 3D-data. Since the purpose of this study is rather to demonstrate the generators abilities than exactly predicting any materials behavior, this simplification does not falsify later conclusions.

#### 3.3.1 Grain properties

**Figure 6:**
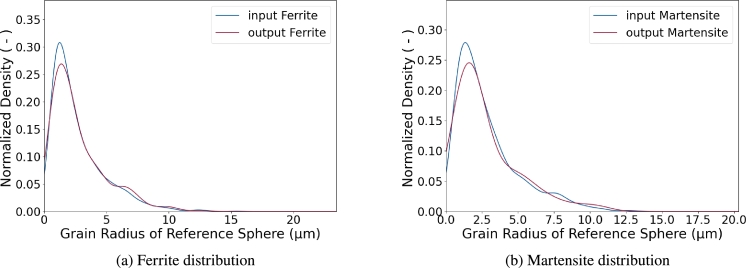
Probability density distributions of the reference grain for EBSD measurement (input) and the RVE (output), separated in the two coexisting phases ferrite (a) and martensite (b).

In order to have a pure comparison of the size, all grain volumes in the experimental data set and the RVE were assumed as spheres and the diameter of these reference spheres was used for the calculation of a Kernel Density Estimation (KDE). Such kind of KDE is shown in Fig. 6. It shows the comparison between the grain size distribution of the EBSD measurements and the RVE. It is clearly visible that the distribution from the EBSD measurement is in very good agreement with the results from the RVE. Therefore, the RVE represents the grain size distribution of the microstructure sufficiently.

**Figure 7:**
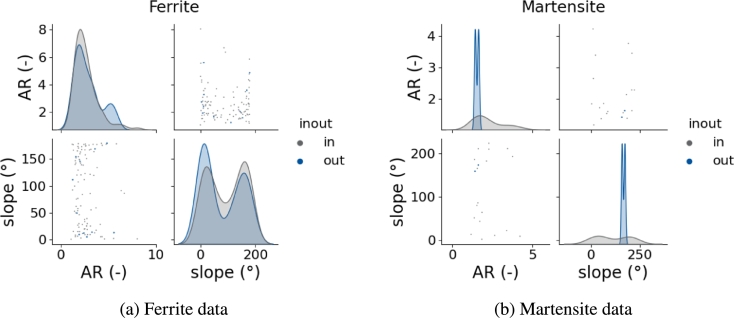
Comparison of input and output of grain shapes and the inter dependency with the grain slope, separated in the two coexisting phases ferrite (a) and martensite (b).

As explained above, the grain shape is experimentally determined with ellipse fits and the first and third halfaxis of each ellipsoid match each other. Therefore, only the first and second halfaxis can be validated with experimental data. This was done by slicing an RVE and performing ellipse fits on each slice. From these ellipse fits the ellipse axis *a* and *b* as well as the slope of each ellipse were analyzed and compared to the experimental data. The comparison is shown in Fig. 7.

It was argued above that not only the distribution for each features themselves is important but also the reconstruction of the interdependencies. In the given example a dual phase steel was reconstructed with a ferritic and martensitic microstructure. It is important to analyze each phase separately in order to not mix characteristics of different phases. The pairplots in Fig. 7 show for both phases that the data extracted from the ferrite phase of the RVE follows the experimental data very well and even more important, no implausible grains are being reconstructed. Also, the shape of the distributions agrees well. The martensite phase seems to be in bad agreement at first sight. However, it must be considered that the data was only drawn from 100 grains and the peaks appearing in the output data are exactly where peaks are located at for the input data. Therefore, it can be assumed that the difference in the KDEs comes from the small amount of data only, is however also representing the material properly.

#### 3.3.2 Phase fraction

**Figure 8:**
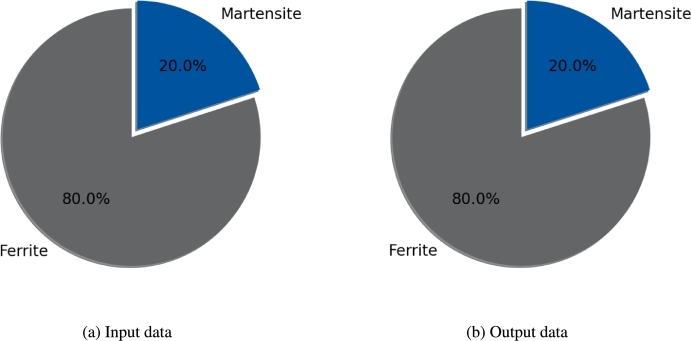
Comparison of desired input phase fractions (a) and output phase fractions (b).

In addition to the grain related distributions, phase fractions must be considered as well. The comparison of experimental data and reconstructed data is shown in Fig. 8. In this case the experimental value for each fraction has to be inserted by the user manually. This allows the user to change the phase fraction easily to other values if needed for investigations. In the given case a ratio of 80/20 of ferrite and martensite was chosen. Again for the phase fraction the output agrees well with the input data, supporting the representativeness of the model even further.

#### 3.3.3 Inclusions and pores

**Figure 9:**
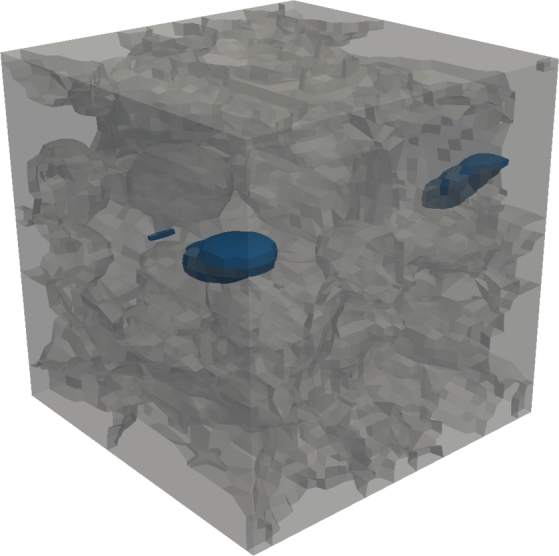
Shown in gray the grain boundaries of the metallic phase and in blue all non metallic inclusions/pores in the RVE.

Another feature in metallic microstructures is the presence of non metallic inclusions and pores. In the current versions inclusions can only be placed into grains. They are placed as ellipsoids, using negative grain IDs starting at −201, after the tessellation process to make sure grain boundaries are being avoided. Inclusions that are located on grain boundaries are currently being worked on and will be part of future versions as well. In order to add inclusions or pores the same type of data is needed as for any other phase. However, no crystallographic orientations are applied to the material. In case the RVE is generated for an FE-Solver and pores are required the elements assigned with the pore will simply be deleted from the model. Fig. 9 shows an exemplary built RVE with inclusions, where the inclusions are visualized in blue and the other phases are transparent. The inclusion/pore feature can also be used for the analysis of microstructures with an initial damage condition as it appears in components that had to go through a forming process [46].

#### 3.3.4 Banded structures

Some materials such as the Dual Phase steel of the grade DP800 show banded structures. The mechanical and microstructure properties of the DP800 discussed in this study have been discussed by Pütz et al. [47] before. The microstructure of this material is composed of ferrite and martensite, with martensite content about 32% of the volume.

**Figure 10:**
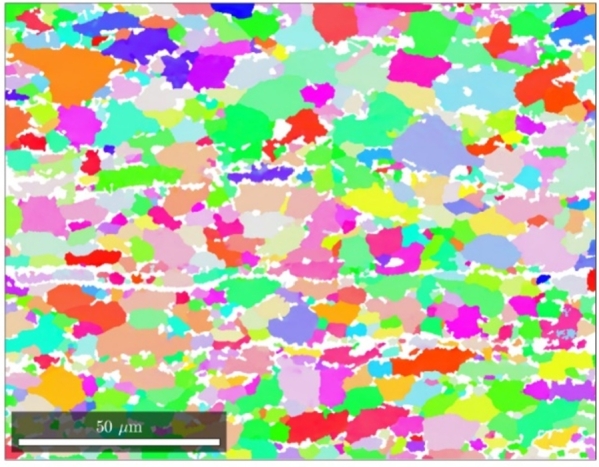
EBSD image of DP800 microstructure. Ferritic phase shown in orientation colormap, martensitic phase in white.

**Figure 11:**
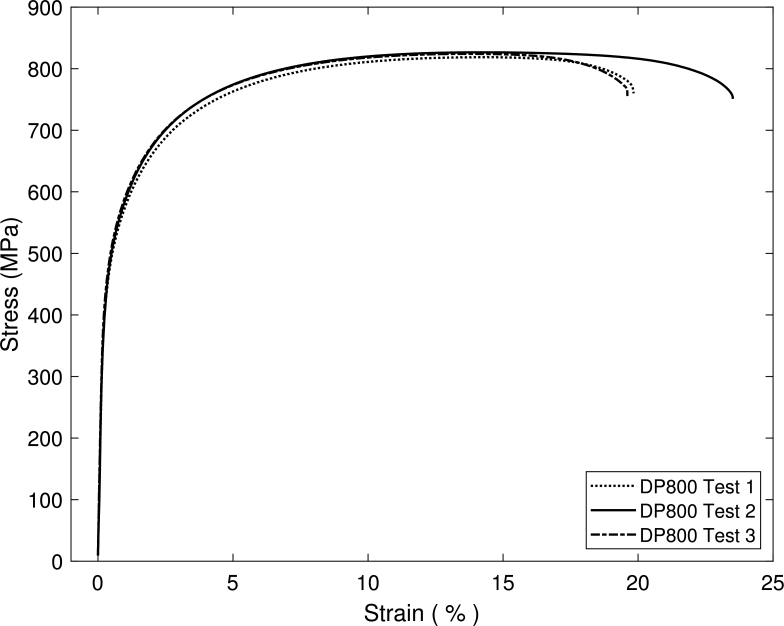
Engineering stress strain curve of uniaxial tensile tests for DP800 for three different tests shows a certain scatter of the damage behavior. Data used from Pütz et al. [47].

An EBSD image of the microstructure in the TDxND-plane is shown in Fig. 10. The martensitic phase, visible in white, shows a banded structure. According to Pütz et al. [47] martensite bands have a significant influence on the damage behavior which is why it is of high interest to properly reconstruct this banded structures and analyze their behavior in a numerical approach [48], [49]. The mentioned scatter in failure can be observed in the engineering stress strain curve in Fig. 11.

**Figure 12:**
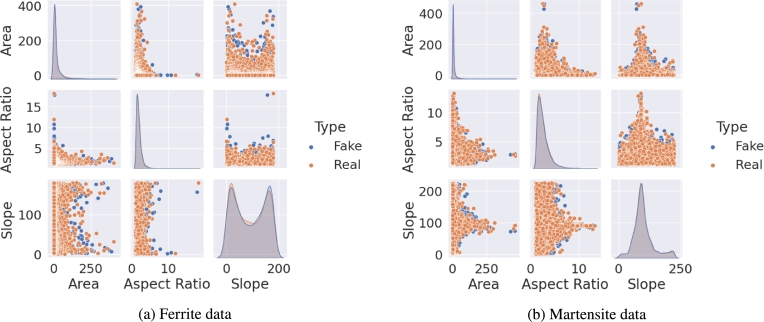
Pairplots showing training data and synthetic data of the ferrite (a) and martensite (b) phase in DP800 steel. Data used from Pütz et al. [47].

Furthermore, the microstructure analysis was taken from Pütz et al. [26] since the same material was discussed in this study. The training and synthetic data which was used for the microstructure reconstruction is shown in Fig. 12a and Fig. 12b.

**Figure 13:**
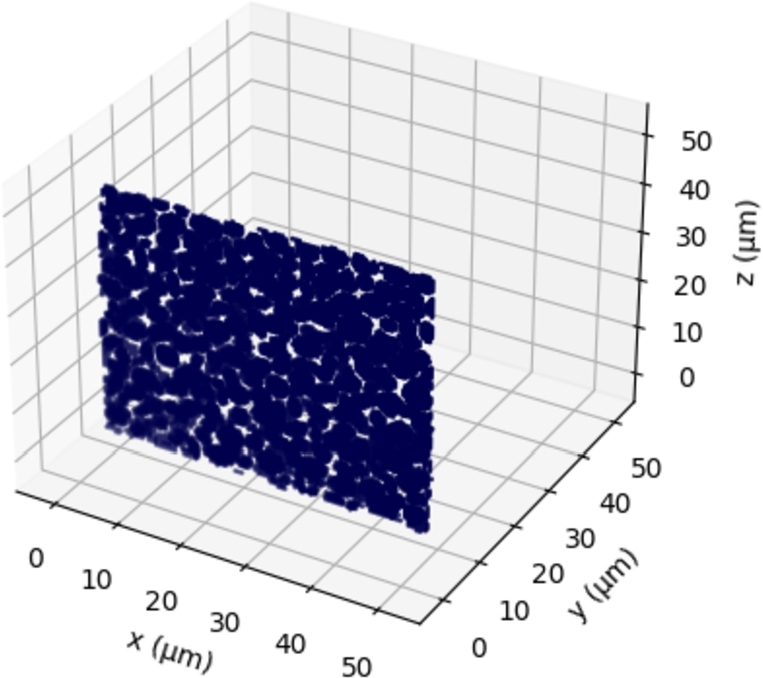
Final stage of banding DRSA with martensite ellipsoids filled in band region only.

In order to generate a banded martensitic structure, an input file for the martensitic phase is needed together with some information about the banding. The user has to define a number of bands for the RVE as well as a maximum and minimum thickness of each band. The generator will then define a cuboid volume for each band defined by the user. The thickness of each band is randomly chosen between the minimum and maximum value given by the user. Also, a band filling parameter has to be defined. This value defines the volume fraction of the cuboid volume that has to be filled with martensite. The cuboid volumes are filled with the ellipsoids from the input file with the DRSA logic. It is therefore called banding DRSA. A visualization of this step is shown in Fig. 13.

Thus, the band filling parameter is responsible for the martensite content within one band. Band filling =1 means the band cuboid volume is completely occupied by martensite. Band filling =0 means there is no martensite at all in the cuboid volume. Neither one of these values is a reasonable choice. The band filling parameter is assumed to be somewhere between 0.4 and 0.65. The theoretical upper limit lies somewhere at around 74%, since this is the highest packing density for identical spheres. However it was found that the generation process is drastically slowed down when choosing values higher than 60% to 65%.

After the necessary martensite volume fraction defined by the band filling value is reached the banding DRSA is stopped, the regular DRSA takes place and the regular generation continues. The martensite ellipsoids in the bands are treated as regular grains in the following generation process and are also considered during the tessellation. Only one exception is made for them in order to be able to visualize the band as one, at the very end of the generation process all material points belonging to a martensite band are indexed with the same grain ID which is −200. That way the filtering for the band can easily be accomplished in following parts of the code, e.g. for the material assignment when generating the final microstructure model for simulations.

**Figure 14:**
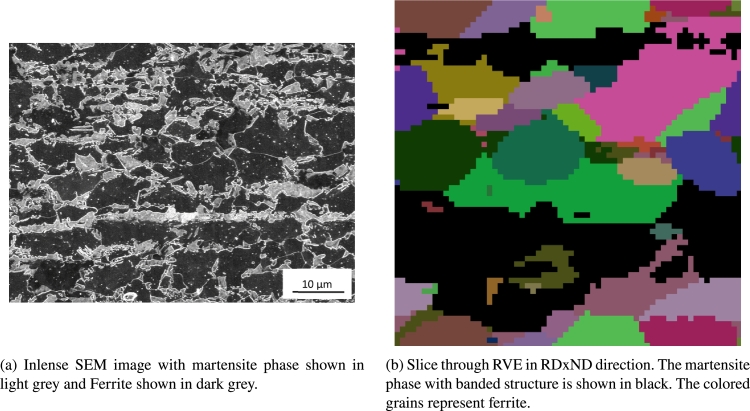
Martensite bands shown SEM image (a) and RVE slice (b).

Due to the lack of 3D-data for the banded martensite structures, the 3D-Structures of the martensite bands can only be validated visually by comparing them to 2D-images from LOM measurements in different directions. Therefore, Fig. 14a with an intense SEM image in RDxND direction is compared to an RVE slice in the same direction shown in Fig. 14b. The RVE contains a banded microstructure with a band filling parameter of 0.48.

As already mentioned to validate this parameter in a trustworthy manner 3D-data is necessary. What can be said however, is that these structures fit the 2D-data very well. Since images were taken from all three directions and the RVEs fit the 2D-data in all three directions, it is reasonable to assume that the 3D-data were also reproduced very well. Therefore, the presented technique is considered an excellent approach for the band reconstruction. The validation with real 3D-data will be part of future studies with experimental results of serial sectioning using a Plasma-FIB and EBSD images.

#### 3.3.5 Crystallographic texture

As an example material for pronounced crystallographic textures a non oriented electrical steel with 0.3 mm thickness (NO30) is presented here. This material is well suited for a first attempt to reconstruct the texture, since it holds a pure ferritic microstructure with relatively simple grain shapes that are near to circular or spherical if three dimensions are considered. Therefore, no influences from secondary phases or complex grain shapes are expected. For microstructure analysis, samples of the material were etched and investigated with LOM and also EBSD pictures were taken.

**Figure 15:**
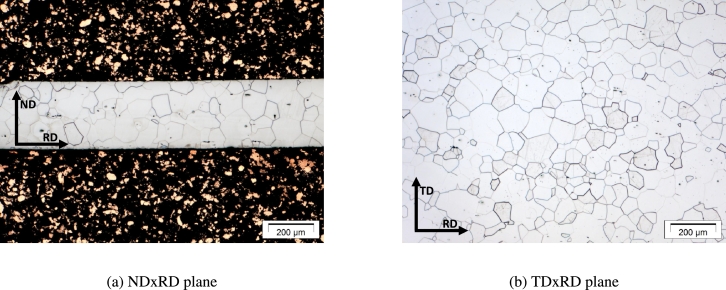
Microstructure of electrical steel under LOM in different directions, NDxRD (a) and TDxRD (b).

Fig. 15 shows the ferritic microstructure of the material in two different planes. The Normal Direction vs. Rolling Direction plane (NDxRD) is shown in Fig. 15a and the Transversal Direction vs. Rolling Direction plane (TDxRD) is shown in Fig. 15b. From these two figures the assumption was drawn that the grains in the material can be described by spheres since there is no direction in which the radius of the grains behaves different. This assumption also makes the necessity of a third picture in the TDxND plane obsolete as well as EBSD measurements in more than one plane.

**Figure 16:**
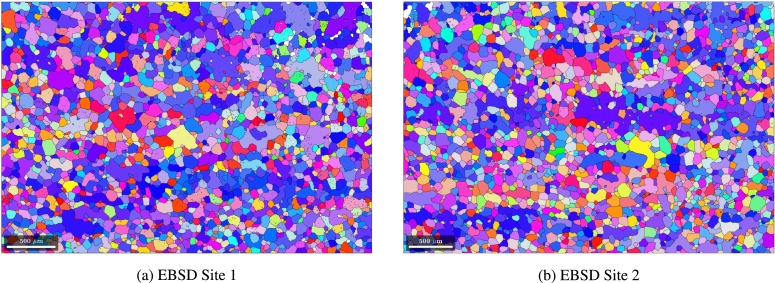
EBSD measurement of the electrical steel microstructure in TDxRD plane at two different locations (1 (a) and 2 (b)).

**Figure 17:**
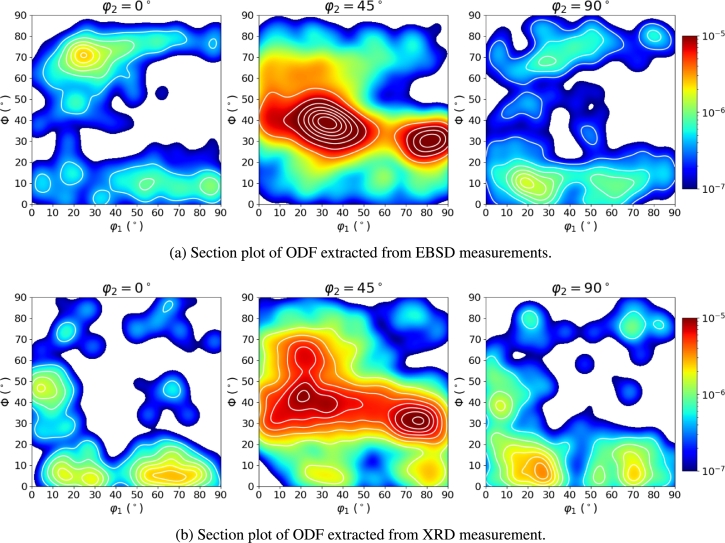
Comparison of texture analysis from EBSD (a) and XRD (b) measurements.

Fig. 16a & 16b show the EBSD measurements performed on two different locations of the material in TDxRD plane. The grain calculation was performed with MTEX (version 5.6.1) [33]. For the grain identification an angle deviation of 10^∘^ was chosen. This is a rough estimation for large angle Grain Boundaries (GB) which were identified as obstacles for dislocation movement in microstructures by Zhang and Wang [50] and Zhang et al. [51]. They state that large angle GB are likely to be an obstacle for dislocation movement and persistent slip bands which occur during cyclic loading. Low angle GB however, are transparent to this kind of effect. Therefore, low-angle GB are neglected in this study and only the large-angle GB are considered. Each calculated grain is plotted with its mean orientation. From the orientation data of these two EBSD measurements the Orientation Distribution Function (ODF) could be calculated and plotted in section plots as shown in Fig. 17a.

For better comparability with the resulting data of the RVE generator the ODF calculation was done in python by calculating the Kernel Density using a gaussian kernel. This is a similar approach as it is suggested for the texture analysis in MTEX [33]. By checking these plots a certain distribution of the orientation angles is visible also in the section plot for $\phi_{1}=45^{\circ }$ a preferred fiber becomes visible. The texture seems to be a mixture of the *α*-, *θ*- and *γ*-fiber [52]. The A-Parameter which is often used for the evaluation of magnetic behavior in electrical steel is calculated as A=33.54 while the texture index t=6.50. The calculation of these two parameters was done in Matlab using the MTEX toolbox and equations (1) & (2) [52], [33].

(1) $$A=A_{\theta }\text{ d}\theta$$

with

(2) $$A_{\theta }=\int f(g)A_{\theta }(g)\text{ d}g\;t=\int f{\left(g\right)}^{2}\text{ d}g$$

Equation (1) is the definition of the A-Parameter, where $A_{\theta }$ represents the orientation averaged value of $A_{\theta }(g)$, which is defined as the minimum angle between the magnetization vector M and the closest 〈100〉 direction. The term f(g) represents the ODF of the material in dependence of the rotation *g* [52]. The texture index in Equation (2) on the other hand is a characteristic scalar which quantifies the pronunciation of the preferred texture orientation, meaning the higher the texture index value the more pronounced the preferred orientation [53].

In total the two EBSD measurements shown in Fig. 16 contain 4374 grains considering the above mentioned constraints for grain boundaries. In order to make sure this is a statistically representative measurement an additional X-Ray Diffraction (XRD) measurement with 72310 data points was performed to get some better statistics about the texture. The section plot of the XRD measurement is shown in Fig. 17b.

**Figure 18:**
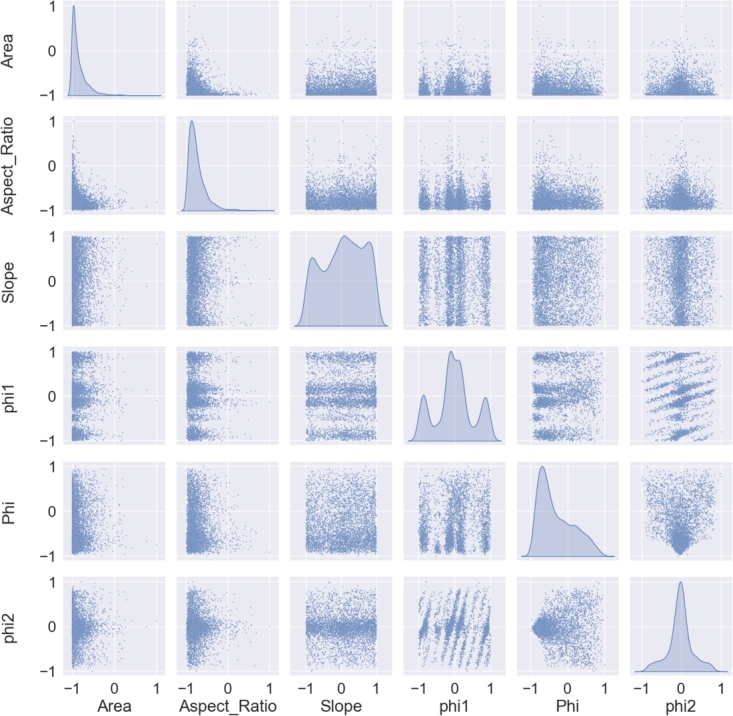
Trainig data for microstructure reconstruction extracted from EBSD measurement.

The difference in the ODFs clearly results from smaller statistics in the EBSD measurement. However, the regions showing high probability densities in the EBSD ODF and the XRD ODF agree well. Thus, the orientation data from the EBSD measurement is considered to show reasonable representativeness. Therefore, the information extracted from this EBSD measurement will be used as training data for the microstructure reconstruction algorithm introduced by Pütz et al. [26]. In this study the modified version of the algorithm will be used which is presented by Fehlemann et al. [27]. The training data for the microstructure reconstruction algorithm is shown in Fig. 18.

**Figure 19:**
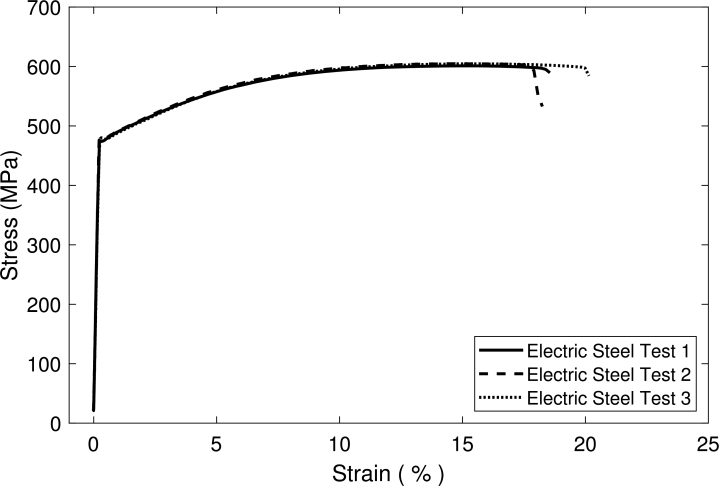
Stress strain curves from three tensile tests performed on samples transverse to rolling direction.

Finally tensile tests were performed for iterative CP-Parameter Calibration. These tests were performed with the loading direction along the transverse direction of the material. Fig. 19 displays technical stress strain curves resulting from these tensile tests.

The plain modeling of the texture in the current generator version is rather simple and is fully dependent on the input data. As shown in Fig. 18, the crystallographic texture can be set in dependence of any other microstructure parameter and therefore it is possible for the WGAN algorithm to generate a set of grains which follow the exact same pattern as it is observed in the training data set. The synthetic data then contains all the information needed to generate a pronounced texture within the RVE. This means the crystallographic orientation of each grain is already defined in the input data and will not be changed any more during the generation process. In other words, the crystallographic orientation is coupled to the grain-ID which is fixed even before placing the grains into the volume. The great advantage of the input data generated with the WGAN algorithm is the certainty that all interdependencies regarding texture and other microstructure parameters are represented correctly. It is however also possible to perform a texture analysis by generating a set of grains that follow a certain orientation distribution as shown in Figs. 17b & 17a, while keeping all other parameters constant. One important aspect, which is currently neglected, is the misorientation of grains. The current method of assigning the crystallographic orientation and placing the grains in the volume does not allow a control mechanism for neighborhood relationships of the orientation in grains. These relationships should however not be neglected in order to make the microstructure model even more realistic. Therefore, this aspect is currently being worked on.

Another feature which was necessary to reconstruct this material correctly was the option to generate non-cubic RVEs. The grains in the NO30 are of such high volume, that cubic RVEs modeled over the complete sheet thickness are still not large enough to reconstruct the texture. Therefore, an option was implemented in the DRAGen code, which offers the possibility to choose a separate size for each direction (x,y,z) of the RVE. This eliminates the periodic modeling of grains in any direction for which a unique size has been chosen, since it is assumed to represent a material surface. It is therefore possible to completely turn off the geometric periodicity by setting a unique size value for all three dimensions, if needed.

**Figure 20:**
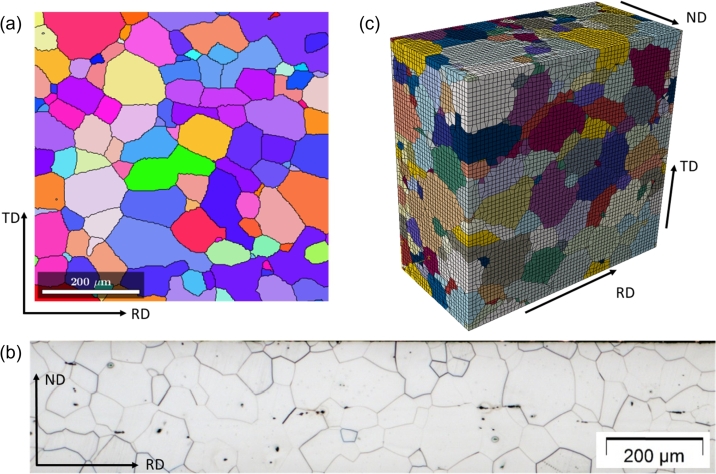
Visual comparison of EBSD measurement (a), LOM image (b) and generated DRAGen RVE (c). The RVE dimensions are 600 μm x 600 μm x 300 μm.

Fig. 20 shows an exemplary RVE of the electrical steel grade. It contains 663 Grains and the dimensions were chosen to be 600 μm x 600 μm x 300 μm. The dimensions were chosen in this way due to the dimensions of the material used for the tensile test, which has a thickness of 300 μm. It was found that an RVE of this material with the dimensions of 300 μm x 300 μm x 300 μm only contains around 100 to 150 grains, which is not enough to represent the texture. Therefore, the thickness was kept at 300 μm and the width and length was extended to 600 μm which led to a sufficient amount of grains.

**Figure 21:**
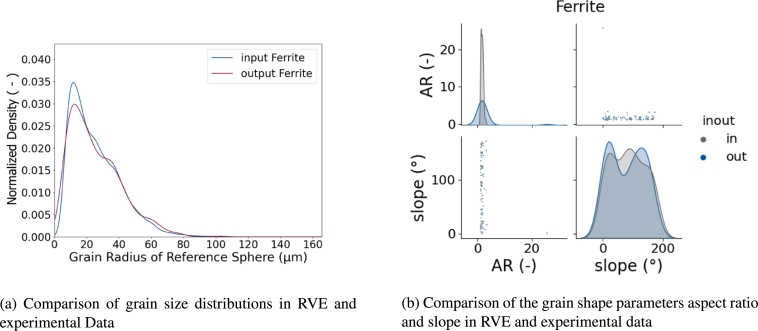
Grain size (a) and shape (b) distribution of the RVE in comparison with the experimental input values.

Also directly in Fig. 20 an EBSD image with similar dimensions and an LOM image are shown for better comparison. It can be seen that the grain shape and grain sizes already look very similar in the experimental images and the RVE. For a more quantitative analysis in Fig. 21 the grain size (Fig. 21a) and shape (Fig. 21b) distributions are depicted. The comparison shows very good agreement on all levels, allowing the statement that the materials morphology is sufficiently reconstructed for a proper microstructure analysis using numerical methods.

**Figure 22:**
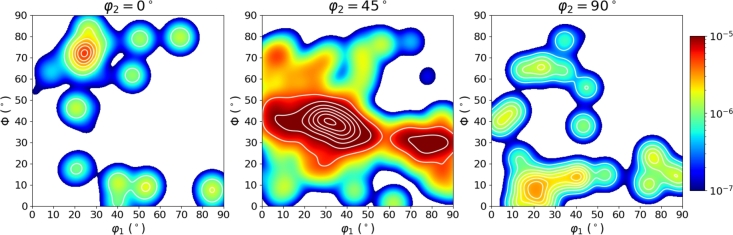
Section plot of ODF extracted from the RVE.

The key feature of the electrical steel, the pronounced texture, is investigated by plotting the ODF as shown in Fig. 22. It was extracted from the final RVE shown in Fig. 20. Compared to the section plot in Fig. 17a this is a very promising result. The A-Parameters and the texture index were calculated to be A=33.8 and t=4.8.

From Table 3 and Fig. 22 the conclusion can be drawn that the number of grains is clearly the limiting factor in texture reconstruction. While the A-Parameter is represented near to perfect by the RVE the texture index *t* is a bit inaccurate. Yet, the difference between the ODF of the EBSD and the ODF of the XRD is much bigger than the difference between RVE and EBSD. Meaning the reconstruction worked properly, is however highly dependent on the number of grains.

**Table 3:** A-Parameter and Texture index calculated from XRD, EBSD and RVE.

	A	t
XRD	32.5	2.8
EBSD	33.5	6.5
RVE	33.8	4.8

#### 3.3.6 Hierarchical substructures

**Figure 23:**
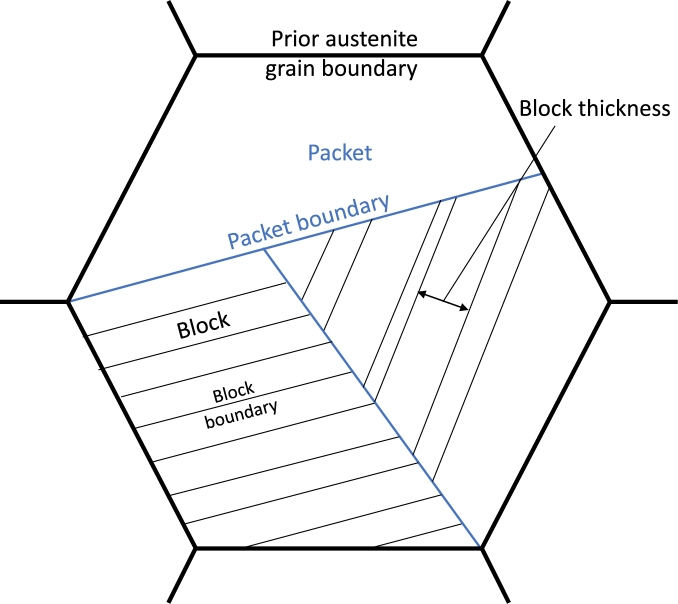
Schematic illustration of the hierarchical substructure of martensitic and bainitic steels according to [11].

Martensitic and bainitic steels, have a microstructure feature in common, which is called hierarchical substructure in the DRAGen RVE generator. This feature contains certain relationships between Prior Austenite Grains (PAG) and the subsequently forming martensite or bainite phase, respectively. These relationships are called orientation relationships and can be used to reconstruct the crystallographic orientation of the parent phase which is for both, martensite and bainite the austenite phase. [13] The substructure found in martensitic and bainitic materials follows a certain hierarchy which is schematically shown in Fig. 23.

**Figure 24:**
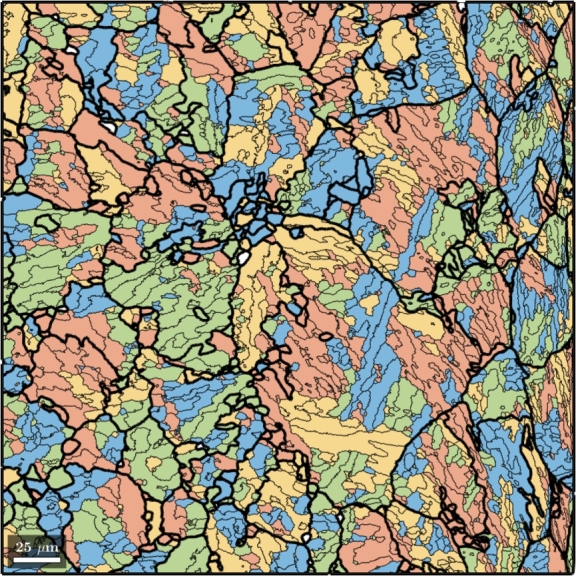
EBSD-Map with reconstructed PAGs (bold black lines), Packets (red, green, yellow and blue areas) and Blocks (thin black lines) created with MTEX.

In this study a bainitic steel was investigated with EBSD measurements. These measurements were then analyzed with the Matlab toolbox MTEX and parent grains could be reconstructed. The reconstruction was performed as suggested in the MTEX documentation and published by Niessen et al. [13]. This reconstruction approach mainly relies on Kurdjumov-Sachs, Nishiyama-Wassermann relationships, respectively [54], [55]. The result of the reconstruction is shown in Fig. 24. In the case of this study Kurdjumov-Sachs relationships were used.

The bold black lines represent the PAG boundaries while the thin black lines are block boundaries. Blocks with the same color belong to the same packet.

For the reconstruction of bainitic or martensitic materials that follow hierarchical substructures an additional module was implemented which takes care of the substructure generation after a model with grains representing the PAGs has been fully tessellated. For this purpose the PAG representing volumes are processed with two further algorithms the first one is a Binary Space Partitioning (BSP) algorithm which is responsible for the generation of packets and the second one is responsible for the generation of blocks and their crystallographic orientation within each packet.

**Figure 25:**
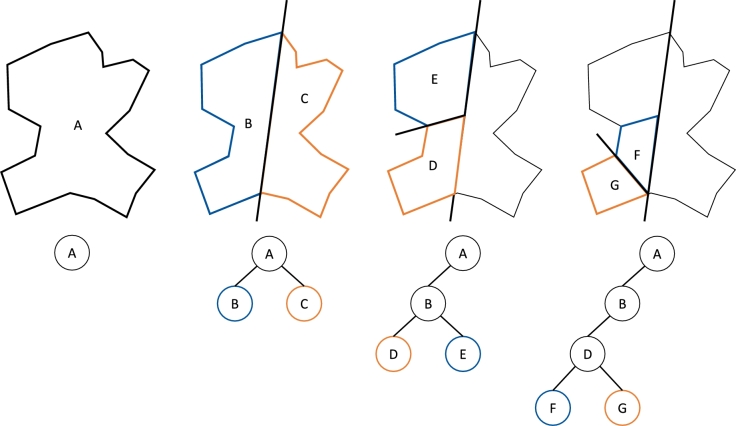
Schematic illustration of the BSP algorithm.

The BSP algorithm is a recursive, hierarchical partitioning of d-dimensional space by (sub)hyperplanes. It is a widely used algorithm in computer graphics and has proven to be an efficient algorithm for many tasks, such as polyhedron representation, set operations, point classification, etc. [56]. The partitioning process by BSP can simply be understood as a process that takes a subspace and partitions it by a hyperplane that slices through the interior of that subspace. This partitioning creates two more new subspaces that can be further partitioned. The process of partitioning in 2D-space is shown in Fig. 25. The subspaces can be seen as the space on the right and left of the hyperplanes. Mathematically this description is formulated in Equation (3) & (4):

(3) $$H^{+}=\{\left(x_{1},x_{2},x_{3}\right)|Ax_{1}+Bx_{2}+Cx_{3}+D>0\}$$

(4) $$H^{-}=\{\left(x_{1},x_{2},x_{3}\right)|Ax_{1}+Bx_{2}+Cx_{3}+D<0\}$$

$H^{+}$ defines the space on the right of the hyperplane and $H^{-}$ the space on the left, $x_{1},x_{2},x_{3}$ are cartesian coordinates of the space and in the case of DRAGen they can be seen as the coordinates of each grid point. The parameters A,B,C and *D* are parameters that define the orientation and position of the plane in 3D-space. The plain logic of the BSP algorithm is shown in Algorithm 1.

**Algorithm 1:**
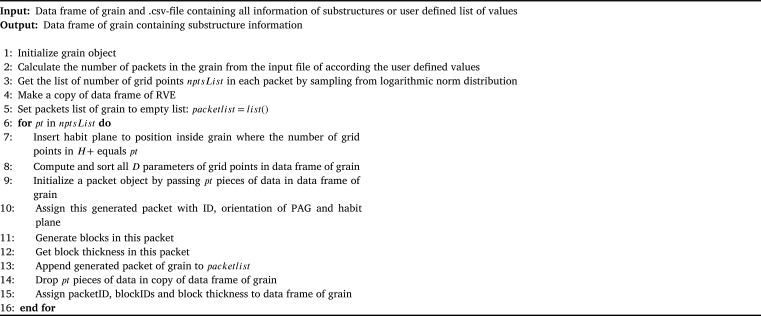
Binary Space Partitioning if Grains into Packets.

Because the block boundaries are parallel, a random norm direction **n** for these parallel block boundaries is chosen for each packet. Then a series of block thickness $bt_{1},bt_{2},...,bt_{n}$ is sampled from a lognorm distribution, in which the $bt_{i}$ are the distances between adjacent martensite blocks. Afterwards, these block boundaries can be represented by P(x,y,z):Ax+By+Cz+D=0, where the coefficients *A*, *B*, and *C* are the components of the chosen norm direction n(A,B,C).

The blockIDs of grid points are assigned based on their distances to a reference plane P(x,y,z). Here, the reference plane is the plane with maximum *D* value $P(x,y,z):Ax+By+Cz+D_{max}=0$. The distances can be computed by the accumulative sum of block thickness list $bt_{1},bt_{2},...,bt_{n}$. Then the distances list $d_{1},d_{2},...,d_{n}$ can be obtained, in which $d_{i}$ denotes the distances of block boundaries to this reference plane and $d_{i}$ is calculated as shown in Equation (5):

(5) $$d_{i}=\sum_{i=1}^{n}bt_{i}$$

Then the blockIDs of the grid points can be assigned by Equation (6):

(6) $$blockID=i\;\text{if}\;d_{i}<d<=d_{i+1}$$

in which *d* is the distance of the grid point to the reference plane.

After the packets have been generated with the BSP algorithm blocks with the assigned block thickness must be generated and a crystallographic orientation has to be assigned to each block. This procedure is implemented in a second python module with the logic as shown in Algorithm 2. In this module it is distinguished between the input data coming from a user defined file or a user defined distribution function for the block thickness.

**Algorithm 2:**
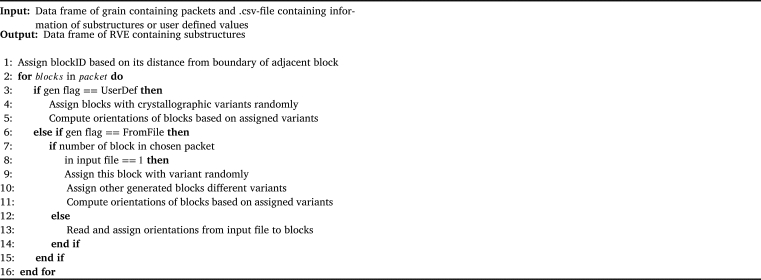
Blocks generation and computation of crystallographic orientation.

**Figure 26:**
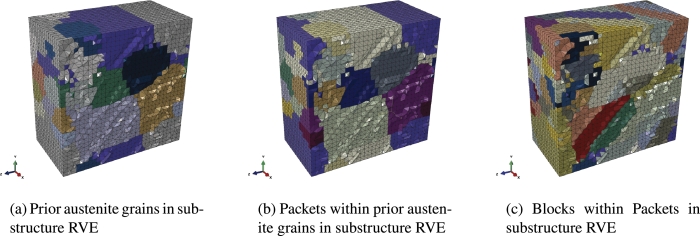
View on the interior of an RVE with hierarchical substructures, showing PAGs, Packets and Blocks which were modeled according to experimental data and the Kurdjumov-Sachs relationships.

Fig. 26 shows an exemplary RVE generated with the input data containing a hierarchical substructure. The figure illustrates all three levels of the substructure that can be accessed within Abaqus. The PAGs shown in Fig. 26a, the packets in Fig. 26b and blocks in Fig. 26c. The model generated with DRAGen is therefore already more detailed than any RVE that can be generated common RVE generators.

**Figure 27:**
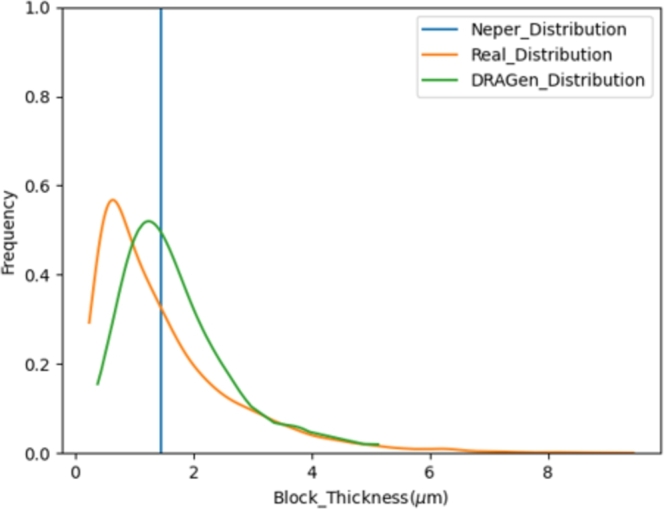
Comparison of the Blockdistribution in DRAGen model, Neper models and experimental data.

Also, DRAGen supports a block thickness distribution following a Lognormal-distribution. Fig. 27 shows the block thickness distribution of the EBSD data and of RVE data. Although, the block thickness distribution of the DRAGen RVE does not perfectly suit the experimental data, there is still a certain representation of the change in block thickness while other RVE generators rather neglect these distributions on lower levels. Therefore DRAGen delivers models closer to reality than RVEs from other generators. The blue line representing the Neper model in Fig. 27 emphasizes this statement, since the values for Neper do not have any variation. This clearly shows the lack of information in models generated by Neper. In terms of distributions Nepers block thickness can be seen as a *μ*-centered normal distribution with a variance of σ=0, while *μ* is the mean value of the block thickness or simply as the Dirac delta function shown below in Equation (7) [57], [58].

(7) $$\int_{-\infty }^{\infty }\delta (\mu )d\mu =1$$

## 4 Application of DRAGen v.1.0

In the following section simulation results are presented which come from simulations using DRAGen RVEs incorporating some of the above mentioned features. Also, all three frameworks were used to conduct these results in order to show the generators flexibility and capability to generate models which can directly be used for simulations with all of the mentioned frameworks. The crystal plasticity models used in each framework differ slightly are however build on the same basis. All three models use a phenomenological approach where the critical shear stress $\tau_{c}^{\alpha }$ is calculated using the shear rate ${\overset{˙}{\gamma }}^{\alpha }$ and the resolved shear stress $\tau^{\alpha }$. The basic mathematical formulation for these kind of crystal plasticity models was developed by Rice [59], Hutchinson [60] and Peirce et al. [61], [62]. Their work led to the following equations (Equations (8) - (12)) for the kinematic behavior of a fcc crystal on the slip system *α*:

(8) $${\overset{˙}{\gamma }}^{\alpha }={\overset{˙}{\gamma }}_{0}{\left|\frac{\tau^{\alpha }}{\tau_{c}^{\alpha }}\right|}^{\frac{1}{m}}sgn(\tau^{\alpha })$$

The parameter ${\overset{˙}{\gamma }}_{0}$ and *m* are material parameters representing the reference shear rate and the slip rate sensitivity. The influence of any other slip system *β* on the hardening of the slip system *α* is:

(9) $${\overset{˙}{\tau_{c}}}^{\alpha }=\sum_{\beta =1}^{n}h_{\alpha \beta }\left|{\overset{˙}{\gamma }}^{\beta }\right|$$

where $h_{\alpha \beta }$ resembles the hardening matrix:

(10) $$h_{\alpha \beta }=q_{\alpha \beta }\left[h_{0}{\left(1-\frac{\tau_{c}^{\beta }}{\tau_{s}}\right)}^{a}\right]$$

Equation (10) describes the micromechanical interaction between different slip systems in an empirical manner. The parameters $h_{0}$, *a* and $\tau_{s}$ are hardening parameters while $q_{\alpha \beta }$ represents the latent hardening matrix with the value 1.0 for all coplanar slip systems *α* and *β*, and 1.4 for all other combinations.

In order to use these equations for a bcc crystal an additional term is needed for the critical resolved shear stress $\tau_{c}^{\alpha }$. It is changed to $\tau_{c,bcc}^{\alpha }$ defined as:

(11) $$\tau_{c,bcc}^{\alpha }=\tau_{c}^{\alpha }+a^{\alpha }\tau_{ng}^{\alpha }$$

with $a^{\alpha }$ being a coefficient representing the net effect of the nonglide stress on the effective resistance and $\tau_{ng}^{\alpha }$ represents the resolved shear stress on the nonglide plane with the normal ${\overset{˜}{n}}^{\alpha }$ given as [19]:

(12) $$\tau_{ng}^{\alpha }=S\cdot \left(m^{\alpha }⊗{\overset{˜}{n}}^{\alpha }\right)$$

These basic formulations were varied and extended over time but their core can still be found in most phenomenological constitutive material models for crystal plasticity. The variation of each model used in this study can be found in the following publications: [44] (Abaqus UMAT), [22] (DAMASK) and [63], [64] (MOOSE).

**Figure 28:**
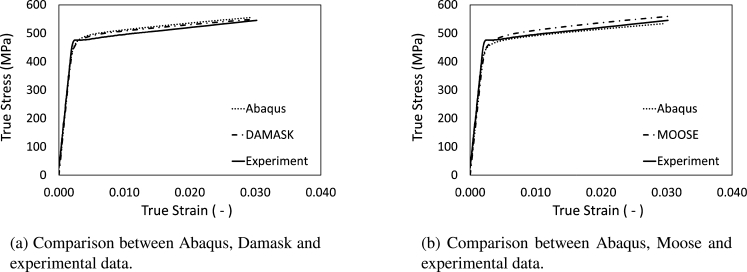
Comparison between different Simulation frameworks Abaqus vs. Damask (a), Abaqus vs. Moose (b) and experimental data.

For the electrical steel, models for all three frameworks were generated in order to show how DRAGen can be used to compare different material models and solvers with the exact same geometrical model. Fig. 28 shows the results of uniaxial tension simulations using a DRAGen RVE generated from the electrical steel data presented in Section 3.3.5. The CP-Parameters were calibrated on the Abaqus UMAT using the RVE as submodel. To keep the models comparable two Abaqus models were generated, one for the comparison with Damask and one for the comparison with Moose. For the comparison with Damask, periodic boundary conditions were applied and for the comparison with Moose, the bottom of the RVE was fixed while the displacement was applied on the top surface, just as it was done in the Moose model. All the simulation results shown in Fig. 28 are in good agreement with the experimental data, yet they also match across the frameworks well. This shows that the transfer of RVEs between the frameworks is possible and well suited for further studies. This supports the statement from the introduction that an accurate modeling approach is highly important to the outcome of a study. So, the possibility of generating the exact same model for multiple solvers and phenomenological approaches gives DRAGen users the capability to investigate the sensitivity of the material models in dependency of quite complex material features. In other words, DRAGen can be used to quantify the sensitivity of each model on the effect of e.g. pores or inclusions or other features such as the texture.

## 5 Conclusion & outlook

In this study a major enhancement to DRAGen v.0.1 published by Henrich et al. [25] is presented. The developments include a revision of the existing code and an improvement of its performance. Additionally, a mesher module for FE-models was implemented as well as new microstructure features such as inclusion and pores, martensite banding, texture representation and hierarchical substructures. It is shown that all of these features can be used to reconstruct certain characteristics of experimentally measured microstructures and comparisons are made with available generators where possible. It is also successfully shown that the DRAGen generator is capable of generating models for three different multiphysics frameworks, namely Abaqus, DAMASK and MOOSE. This offers a high amount of flexibility to researchers using DRAGen for microstructure modeling. The flexibility to choose the framework best suiting a problem best and still rely on the high degree of representativeness ensured by DRAGen RVEs cannot be underestimated. Also sensitivity studies of the different frameworks and a comparison of them is now possible.

The study also showed that DRAGen provides a flexible environment which can be expanded by any feature of interest as it was done here e.g. for the substructure or the texture. This allows future researchers to easily implement their very own features into the microstructure models. If for instance other geometrical shapes than ellipsoids or spheres were needed to reconstruct microstructural features, this could easily be realized with another python module. An option for contributing to the source code is planned to be available via the git repository.

Even though, the results achieved with DRAGen so far are very promising there is still a huge potential for improvement. The texture representation e.g. is still neglecting misorientations which is currently the main focus of ongoing work. Also, in addition to the already existing features, a surface roughness module is currently in the making.

In summary, this RVE generator is well on its way to helping researchers in the ICME community with robust and highly accurate microstructure models, and has the power to provide a solid foundation for future studies in microstructure analysis and design, which in turn will help reducing CO_2_ emissions.

## Funding

The funding of this research by the 10.13039/501100001659Deutsche Forschungsgemeinschaft (DFG, German Research Foundation) – 278868966 – TRR 188, the 10.13039/501100001659Deutsche Forschungsgemeinschaft (DFG, German Research Foundation) – 432053466 and the cluster of excellence “Internet of Production” – 390621612 is gratefully acknowledged. This research is also funded by Federal Ministry of Economics and Climate Protection based on a resolution of the German Bundestag - 22278 N.

## CRediT authorship contribution statement

**Manuel Henrich:** Conceived and designed the experiments; Performed the experiments; Analyzed and interpreted the data; Contributed reagents, materials, analysis tools or data; Wrote the paper. **Niklas Fehlemann, Maximilian Neite:** Analyzed and interpreted the data. **Felix Bexter:** Analyzed and interpreted the data; Contributed reagents, materials, analysis tools or data. **Linghao Kong:** Performed the experiments; Wrote the paper. **Fuhui Shen, Markus Könemann, Michael Dölz:** Contributed reagents, materials, analysis tools or data. **Sebastian Münstermann:** Contributed reagents, materials, analysis tools or data; Wrote the paper.

## Declaration of Competing Interest

The authors declare that they have no known competing financial interests or personal relationships that could have appeared to influence the work reported in this paper.

## Figures and Tables

**Figure A29:**
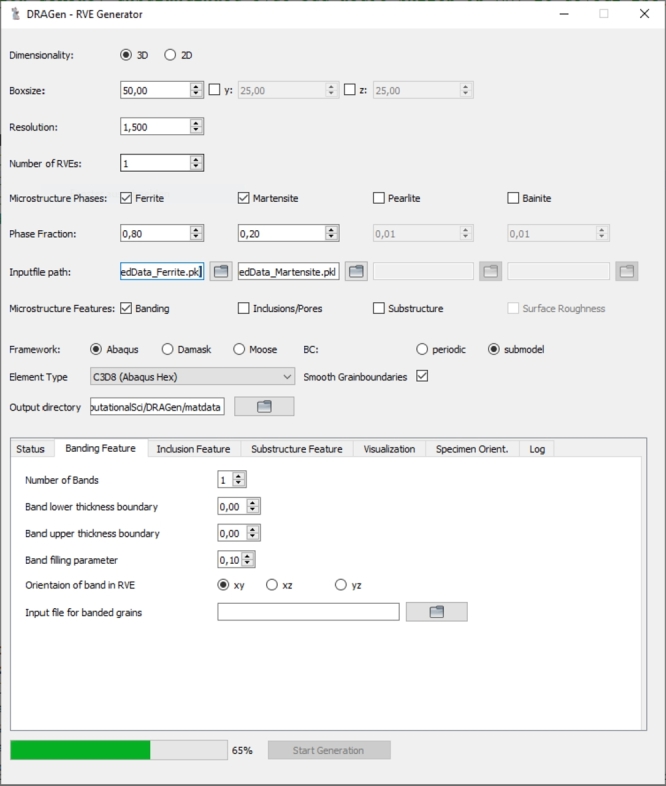
Graphical User Interface of the DRAGen Generator showing the options for the banding feature.

**Figure A30:**
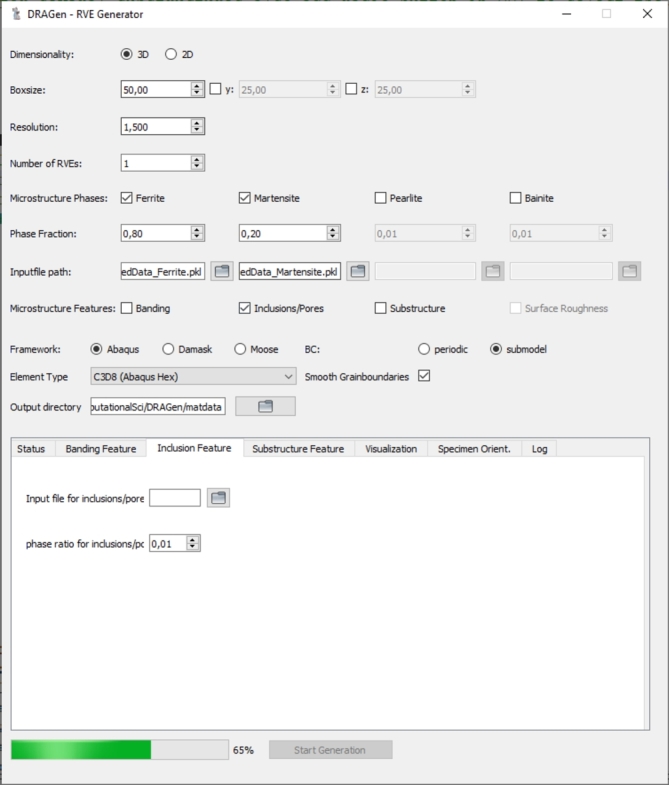
Graphical User Interface of the DRAGen Generator showing the options for the inclusion feature.

**Figure A31:**
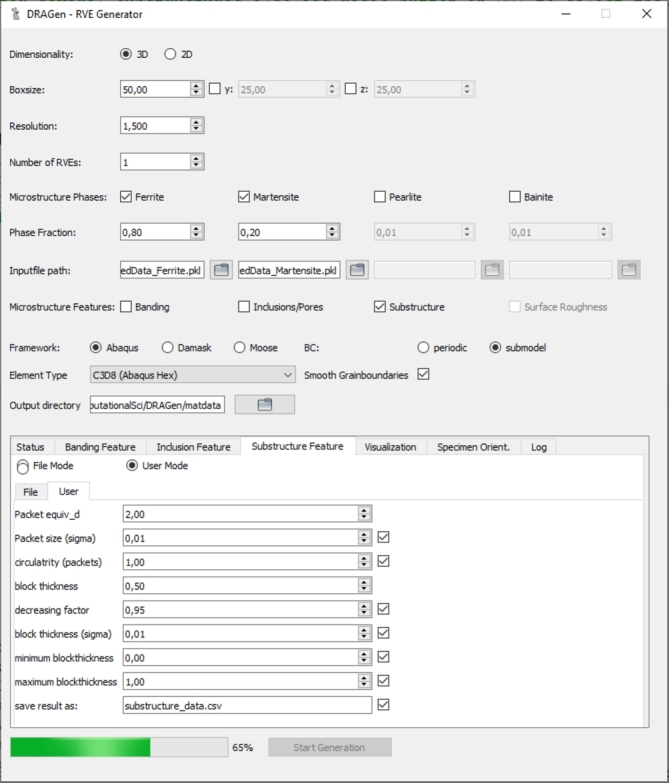
Graphical User Interface of the DRAGen Generator showing the options for the substructure feature.

**Table 4:** Required general parameters in the GUI for RVE generation.

**Parameter:**	**Description:**
Dimensionality	choose between 2D and 3D
Boxsize	edge-length of the cubic RVE in μm
Resolution	number of points on edge equals Resolution x Boxsize
Number of RVEs	if desired the user has the possibility to directly generate several RVEs of the same microstructure. In order to generate RVEs that differ sufficiently from each other the use of synthetic data is highly recommended if this option is used.
Number of Bands	if the microstructure to be reconstructed contains a banded structure in the secondary phase this can be chosen here by setting a certain number of bands that will appear in the generated RVE.
Microstructure Phases	choose the phases for the RVE
Phase fraction	if phase is activated a phase fraction must be defined
inputfile path	if phase is activated a .csv-file or .pkl-file must be given
Microstructure Features	Choose any of the available features
Framework	chose desired simulation framework
Element type	if Abaqus or MOOSE is chosen set the desired element type
Output directory	a path where the generated data shall be stored must be given here. The default path is set to C:/temp.
Features Tabs	depending on the features chosen above the tabs are activated and additional information must be given.

## Data Availability

Data associated with this study has been deposited at https://github.com/ibf-RWTH/DRAGen.

## Appendix A Graphical user interface GUI

Currently there is a prototype of a GUI available for the usage of DRAGen. The main window of this GUI is shown in Figure A29, Figure A30, Figure A31. The user is asked to enter several different parameters that are explained in Table 4.
